# Personalising screening of sight-threatening diabetic retinopathy - qualitative evidence to inform effective implementation

**DOI:** 10.1186/s12889-020-08974-1

**Published:** 2020-06-08

**Authors:** P. Byrne, C. Thetford, M. Gabbay, P. Clarke, E. Doncaster, S. P. Harding, for the ISDR Study Group, Simon P. Harding, Deborah M. Broadbent, Paula Byrne, Anthony C. Fisher, Mark Gabbay, Marta García-Fiñana, Marilyn James, Tracy Moitt, John Roberts, Daniel Seddon, Irene M. Stratton, Jiten P. Vora, Paula Williamson, Duncan Appelbe, Ayesh Alshukri, Christopher P. Cheyne, Darsy Darssan, Antonio Eleuteri, Christopher Grierson, Lola Howard, Susan U. Howlin, James G. Lathe, Mehrdad Mobayen-Rahni, Andy Ovens, Christopher J. Sampson, Kate Silvera, David Szmyt, Clare Thetford, Pilar Vazquez-Arango, Amu Wang, Abigail E. Williams, John Collins, Emily Doncaster, John Kelly, Peter Lees, Sandra Lees, Betty Williams, Catey Bunce, Helen Cooper, Vineeth Kumar, Nathalie Massat, Chris Rogers, Alison Rowlands, Gideon Smith, Julia West, Naveed Younis, Ticiana Criddle, Stephanie Perrett, Lisa Jones

**Affiliations:** 1 Institute of Population Health, University of Liverpool, Liverpool, UK; 2 Faculty of Health and Wellbeing, University of Central Lancashire, Liverpool, UK; 3 ISDR Public Involvement Group, University of Liverpool, Liverpool, UK; 4 Eye and Vision Science, University of Liverpool and St. Paul’s Eye Unit, Royal Liverpool University Hospital, Preston, UK

**Keywords:** Qualitative, diabetic retinopathy screening, acceptability

## Abstract

**Background:**

Internationally, systematic screening for sight-threatening diabetic retinopathy (STDR) usually includes annual recall. Researchers and policy-makers support extending screening intervals, citing evidence from observational studies with low incidence rates. However, there is little research around the acceptability to people with diabetes (PWD) and health care professionals (HCP) about changing eye screening intervals.

**Methods:**

We conducted a qualitative study to explore issues surrounding acceptability and the barriers and enablers for changing from annual screening, using in-depth, semistructured interviews analysed using the constant comparative method. PWD were recruited from general practices and HCP from eye screening networks and related specialties in North West England using purposive sampling. Interviews were conducted prior to the commencement of and during a randomised controlled trial (RCT) comparing fixed annual with variable (6, 12 or 24 month) interval risk-based screening.

**Results:**

Thirty PWD and 21 HCP participants were interviewed prior to and 30 PWD during the parallel RCT. The data suggests that a move to variable screening intervals was generally acceptable in principle, though highlighted significant concerns and challenges to successful implementation. The current annual interval was recognised as unsustainable against a backdrop of increasing diabetes prevalence. There were important caveats attached to acceptability and a need for clear safeguards around: the safety and reliability of calculating screening intervals, capturing all PWD, referral into screening of PWD with diabetic changes regardless of planned interval. For PWD the 6-month interval was perceived positively as medical reassurance, and the 12-month seen as usual treatment. Concerns were expressed by many HCP and PWD that a 2-year interval was too lengthy and was risky for detecting STDR. There were also concerns about a negative effect upon PWD care and increasing non-attendance rates. Amongst PWD, there was considerable conflation and misunderstanding about different eye-related appointments within the health care system.

**Conclusions:**

Implementing variable-interval screening into clinical practice is generally acceptable to PWD and HCP with important caveats, and misconceptions must be addressed. Clear safeguards against increasing non-attendance, loss of diabetes control and alternative referral pathways are required. For risk calculation systems to be safe, reliable monitoring and clear communication is required.

## Introduction

The rising prevalence of diabetes over the past 30 years presents challenging health impacts and costs to individuals, health care systems and wider society. Prevalence rates in the UK rose from 3.2 million people in 2013 to 4.7 million in 2019, and they are expected to rise to 5.5 million by 2030 [1, 2]. Prevalence rates are increasing more rapidly in low and middle income countries [3]. Having diabetes can involve a number of related health issues, including diabetic retinopathy (DR). DR is a major cause of vision impairment among adults worldwide and is the second most important cause of visual loss in England and Wales [4].

In the UK, the National Health Service (NHS) eye screening programmes have offered annual screening to all people with diabetes (PWD) over the age of 12 years for around 10 years. These programmes aim to detect sight threatening diabetic retinopathy (STDR) before it affects a person’s sight and when timely, effective treatment can be provided. Evidence suggests that it may be safe to screen low-risk people at longer intervals [5–11] and the interval has been extended in some countries [12, 13]. However, this evidence is not conclusive and is based largely on modelling rather than experimental research. In those countries, such as the Netherlands, Iceland, and the city of Hong Kong, with extended intervals the population being covered is significantly different to the UK. The shift towards varying screening intervals is not restricted to DR. For breast cancer there are moves to identify risk-stratified screening strategies to lower the rates of over diagnosis and to prevent deaths [14]. Such directions illustrate a general move within medicine to personalised health care and potentially to re-allocate resources to those most in need; in the case of DR screening focusing on non-attenders. Risk estimating equations have been developed to allow this personalisation in DR [15–17] and in other specialties [14, 18]. Nevertheless, there has been little work on the impact on PWD of changing eye screening intervals and concern amongst HCP about safety including reduced attendance and loss of diabetes control [9].

An intervention, such as changing eye screening intervals, can be considered to be implementing evidence-based practice. The aims of an intervention are to promote the uptake and optimal use of effective clinical services, along with modifications to health-related behaviour. It can be anticipated that there may be negative as well as positive outcomes from an intervention, therefore effective development and implementation is essential. Understanding enablers and barriers to change and then putting in place effective strategies to encourage or mitigate against their effect is crucial. Models of behaviour change can be a useful theoretical lens to explore behaviour and how to effect positive change. Such models have been used extensively within clinical and public health arenas to understand illness and health-seeking behaviours [19–21]. There have been moves away from a deficit model, where primarily patients are perceived as lacking in their understanding and simply needing “more education” about their condition to resolve any issues. The Behaviour Change Wheel (BCW) is cognisant of the many components involved in changing health related behaviours, it recognises that the sources for behaviour can be found within three areas and usefully applied to changing eye screening intervals: capability (is the individual able to attend eye screening?); opportunity (does the eye screening service make it as easy as possible to attend an appointment?); and motivation (can an individual manage any changes to their eye screening appointment?) [22]. The BCW approach also offers screening service commissioners and providers a range of interventions and policy approaches to align with PWD and HCP capability, opportunity and motivation to change eye screening intervals. The BCW has been successfully used in a number of other clinical arenas [23, 24].

As part of the ISDR randomised controlled trial (RCT) [25], we undertook a qualitative study with PWD and HCP designed to investigate and uncover enablers of and barriers to behaviour change of moving from annual to personalised risk-based variable-interval screening and to gain wider insights into perceptions amongst PWD and HCP. Our aims were to develop detailed understandings of the acceptability and enablers for, successful implementation of personalised screening in England and other countries with similar systems. We followed Medical Research Council (MRC) guidance on developing and evaluating complex interventions [26].

## Methods

### Setting

A programme of applied research developed an enhancement to screening for STDR by introducing and testing an individualised or personalised approach based on measured patient-centred risk. A novel intervention was developed comprising variable-interval screening determined by a risk calculation engine (RCE) informed by real-time demographic, retinal and clinical data from the individual, referenced to local historical data. Intervals were allocated at 6, 12 or 24 months for high, medium or low risk respectively and recalculated at each screening appointment. A RCT was designed to compare the efficacy and cost-effectiveness of this individualised approach to standard fixed interval screening [25]. A public involvement (PI) group was embedded in the programme. This setting allowed, for the first time, the real rather than theoretical investigation of the enablers and barriers around implementation of varying intervals, and the use of a risk calculator, in a population from an established screening programme and in a geographical location where annual fixed interval screening is already established.

### Design

Semi-structured interviews [27, 28] were conducted to gather views on risk-based variable-interval screening. Interviews with PWD were conducted prior to its implementation (Phase 1, baseline) in the setting of the parallel RCT and subsequently with a second group (Phase 2) during the RCT. All interviews with HCP took place prior to implementation.

The research team and the PPI group created interview topic guides. With PWD these covered understanding about diabetes, self-management, health services contact, responsibility for monitoring diabetes, links between diabetes and eye health and screening intervals. With HCP the guides focussed on diabetes services, current eye screening, future changes to eye screening and DNA rates. All participants received a brief overview of the individualised risk-based variable-interval screening intervention. Most patient interviews were conducted in participants’ homes, though some chose to complete them in the researcher’s university office, and one participant completed their interview in their own work office. HCP were interviewed at their place of work. Interviews lasted between 30 to 90 min, with most lasting around 45 min.

### Participants

PWD aged over 16 years attending the eye screening programme were identified in two General Practices in a city in the North West of England. Suitability for participation was confirmed by a General Practitioner prior to a letter of invitation and patient information pack with reply slip being posted out. PWD who were interested in participating returned the reply slip or contacted the research team to make arrangements for an interview.

Sixty PWD were recruited, 30 to phase 1 (baseline) and 30 to phase 2 (post implementation of risk-based variable-interval screening). Thirty-four of the 60 were men and 8 had type 1 diabetes. The age range was 19–83. Times since diagnosis were: 1–5 years *n* = 10, 6–10 years *n* = 7, 11–15 years *n* = 4 years, > 15 years *n* = 9; range 1–40 years. For phase 2, participant allocation to risk based screening intervals was: 6 months *n* = 4, 12 months *n* = 5, 24 months *n* = 21.

PWD participants reported a range of social situations; the occupations described by the sample were very mixed, including a range of professionals as well as students and manual workers, retired and unemployed as well as one person who was unable to work due to long-term ill health.

HCP were identified by personal and professional local networks of the research team. To help with time commitments HCP could participate in individual interviews, in groups, or by completing an open-ended questionnaire to be returned by email. Interviews were conducted at the HCP’ place of work in a private office (one joint interview was completed at a participant’s home). Most interviews lasted around 40 min. Six of the HCP participants were interviewed as three colleague pairs.

Twenty-one HCP were recruited. Sixteen participated in interviews whilst five elected to complete questionnaires via email. Professional roles were: screener/grader *n* = 7, consultant ophthalmologist (retina specialist) *n* = 5, eye screening service manager *n* = 4, optometrist providing DR screening *n* = 2, public health specialist *n* = 2, general practitioner *n* = 1.

### Analysis

Interviews were audio-recorded and transcribed verbatim to enable detailed analysis. Semi-structured case summaries were produced by a researcher upon completion of each interview to provide a summary of key themes to enable identification of emerging themes to inform further data collection and analysis. After reading and re-reading the transcripts, data were analysed to identify sections of text that informed understandings of the issues [29–31]. Each concept was assigned a descriptive or analytical code, which was then combined into conceptual categories and broader themes. Each of the datasets were coded using NViVO software, which enabled searching and retrieval of specific data.

When key themes were identified, a number of charts were created, based upon the thematic analysis, to enable comparison of the datasets, taking a framework approach [32, 33]. Data from individual participants were entered into cells within the chart to enable comparison on each theme at the level of individual participant and whether they were a PWD or an HCP.

### PI

Our PI group contributed to the research questions and topic guides, informed analyses and further exploration, and one member joined the authorship.

### Ethics

The two phases of the study were conducted under the following research ethics committee approvals: 13/NW/0287 and 16/NW/0061.

## Results

Analysis of interviews with PWD and HCP identified several themes related to changing screening intervals: the acceptability of changing screening intervals in conjunction with conditions and safeguards attached to 6, 12 and 24 month screening intervals; the safety of the RCE; and the macro impact of changing screening intervals.

### Acceptability of changing screening intervals

The majority of PWDs in both Phase 1 and 2 expressed the view that risk-based variable screening intervals were potentially acceptable. The views of PWD included the concepts of pragmatism and diverting any cost savings towards other PWD who may need to be seen more often.

*You know if they don’t need them every year then yes, why do them every year. And so I would rely on the practitioner to make the best judgement.* (David PWD, Phase 1)

*Yes, you know because if your eyes are not going to go any worse it is saving money and time isn’t it, where, they can’t fit everybody in can they….it saves all that money and so it gives other people a chance of getting seen doesn’t it? So, I agree with that.* (Susan PWD, Phase 2)

For HCP, the majority were also in favour of introducing risk-based variable screening intervals.

*I think it will be fantastic because as you say there are lots of patients that you can quite happily review in 2 years, or 18 months 2 years, so I think that would be a definite benefit.* (Sally HCP)

*I don’t have any objections, as long as it is evidence based you know if there is evidence for it being 24 months, then I am fine. If there is evidence for a particular group being 10 years, you know if that what the evidence shows, I am very much things have to be supported by evidence.* (Andrew HCP)

However, implementing such changes to eye screening was accompanied by a range of caveats which are discussed in relation to the 6, 12 and 24 month screening interval. The norm is currently 12 months, so we explore the other options in some detail below, as for some PWD this is more frequent than currently, for others no change, and for many, twice as long.

### Acceptability of 6 month screening interval

Within the variable screening model, allocation to a 6 month interval meant that a PWD was considered to be at high risk of developing STDR. However, for some PWD participants, this rationale was not fully understood, with the shorter screening interval interpreted as a security for checking eyes, with the longer screening interval of 24 months unwelcome as it was seen as too prolonged a time to be without eye screening. Additionally, some PWD viewed screening as a preventive measure in developing DR.

*Well, early detection is better for the treatment you know what I mean, if you find something drastically wrong with your sight, and they can repair it, a lot, and if they find it earlier, they could repair it.* (Arthur PWD, Phase 2)

Some PWDs, notably in the 55 plus age range, wanted to be screened every 6 months, or even every 3 months, and this related to a belief that they were more susceptible to developing eye disease related to diabetes and consequently needed to be monitored more often.

*When you’re over a sort of certain age like say 60 that’s when things start going downhill - 60. I’m not saying like everybody - everyone’s different, aren’t they - but the way I look at it is that I think it should be every six months ... if you’re a type 1 diabetic you should have it every three months ... over a certain age every six months.* (George PWD, Phase 2)

For HCP, the 6 month interval was welcomed unequivocally as a safeguard for high risk patients, and it was stressed that this would have to be clearly communicated to PWD.

*I do like the idea of the medium risk, sorry the high risk ones coming back every 6 months… so I do think that that would be a good thing to have that as a standard if they were high risk, bring them back in the 6 months I think that would be good.* (Sarah HCP)

*…and there certainly are patients who need more closer care, which can’t always be determined just by looking at a picture of their eye ball essentially. You know they may be patients in certain ethnic groups, erm… and certain combinations might, I don’t know, but it might be for example Asian ladies from I am just talking off the top of my head, from a Pakistani origin for example, might it might be part of their societal erm… nature to not necessarily come to attention as much.* (Andrew HCP)

As evidenced above, there are some tensions within PWDs’ understandings about the 6 month screening interval, with it being seen as clinical surveillance which was a reassurance, against the clinical reality that being allocated to this interval means that there is a high risk of developing STDR. Additionally, there was conflation about the purpose of eye screening, where it was commonly perceived to be a preventive measure against DR. For HCP, the shorter interval was welcomed to monitor high risk patients.

### Acceptability of a 12 month screening interval with conditions

As the eye screening service has been in place for over 10 years in England and Wales, it was perhaps unsurprising that some PWD felt that annual screening was acceptable and should remain in place. This was often related to participants’ positive experiences of attending eye screening. Any changes made to their screening interval was felt by some PWD to be up to their HCP to decide upon, as shown below.

*I am quite happy with that. Some people may need close screening but I am quite happy with the 12 months. If they wanted to see me more frequent or less frequent, I would just go along with it.* (Sheena PWD, Phase 1)

The reliance upon HCP to decide which interval to allocate a PWD was mentioned by several of the participants. For other PWD, the annual eye screening appointment was mistakenly perceived as a reassurance and safety net for any changes in the eyes during this time period, illustrating the misunderstanding of the relationship between diabetic health, DR and screening.

*I’d rather be seen every 12 months to be honest. I think people should be seen even if they’re classed as very low risk. I think even within a year a lot can change. You can suddenly have a bout of you know, I don’t know if having a bout of problems with your sugars would affect your eyes but yes, I just think every 12 months it should be*. (Joanne PWD Phase 1)

Whilst some reported feeling reassured by annual screening, there were misunderstandings about the impact of diabetic health upon eye health and the role of screening. As the current annual screening interval is established and embedded into practice, it was foreseeable that PWD felt that this was an appropriate length of time for their eye screening. However, discussions also highlighted misunderstandings about the purpose of eye screening, as a preventive measure against the development of DR, and related to diabetic control.

### Acceptability of 24 month screening interval with conditions

Extending screening intervals to 24 months provoked the most reaction and responses amongst PWD. The range of views included an unequivocal rejection of a 2 year interval as illustrated below.

*No way!* (Melanie PWD Phase 2)

Other PWD were more nuanced in their responses with concerns about this interval being too long a time period to go without being seen within the eye screening programme.

*It’s an awful long time, 24 months, isn’t it?* (Jean PWD Phase 1)

There were allied concerns about this interval around the potential for changes to the eye over 2 years and not being screened.

*I feel like leaving something for two years can be very risky, because someone could always, all of a sudden be in a low risk and then take a turn for the worst and have like their eyes get really bad, really quickly due to something else. I feel like six and 12 months is good but then I don’t think 24 months is good, I think it’s too long because you wouldn’t leave someone who had diabetes for two years and not check their HbA1c, so why would you do it for their eyes?* (Polly PWD Phase 2)

For other PWDs, being assigned to the 2-year screening interval was a positive reflection of their diabetes control.

*I thought well if I don’t need it doing every 12 months then good, send it to two years. And I didn’t think anything bad about it. I suppose I was quite positive about it really. I thought it was working in my favour if it was going to last two years ... not having another appointment to go to ... I always think, if they extend your visits it means you’re on a level playing field you know, things are going smoothly; that’s the way I look at it.* (Mary PWD, Phase 2)

For HCP, the move to 2-year intervals was welcomed as they highlighted that with annual screening they have to screen very large numbers of negative patients in order to identify a screen positive patient and that this seems like an inefficient approach.

*I think looking to change the intervals makes sense to me a lot because an awful lot of the screening we do is, there is nothing there. And, even the ones who have mild background retinopathy you see little or no progression over several years…And you are seeing an awful [lot] of patients who are either having no retinopathy whatsoever or very, very mild retinopathy.* (Gerard HCP)

For HCP, extending the screening interval would enable the better targeting of resources and would benefit patients who, for example are difficult to engage and often do not attend for screening, or who are at higher risk of developing STDR.

*Unless we have the resources to follow all these patients [non-attenders] up, which if we do go to two-years screening we probably would.* (Frankie HCP)

Similarly to PWD, HCP were concerned about extending screening intervals for the potential negative impact upon patient behaviour, namely it would affect risk perception around eye screening attendance. HCP anticipated that some patients would interpret extended intervals to mean that eye screening is considered not essential and there would be a concomitant effect upon an increase in non-attendance.

*If you give someone a two-year appointment, they are probably thinking, well it can’t be that important if I don’t have to come back for two years.* (Sami & Suzanne HCP)

Some HCP drew upon their clinical experiences to support an argument about their unease on extending screening intervals being at odds with their embedded narrative to PWD of needing to be screened annually and related to the trust and relationship between PWD and HCP.

*I just don’t agree with it. We have spent, well I have spent the last 11 years drumming it into patients how important it is to be screened every 12 months, and now this is just going against everything I have been saying. And 12 months is a long time, and serious, serious damage can happen in them 12 months, even if they have had nothing in the past, I have seen it so many times. So I just don’t think it is worth the risk of moving a patient to 24 months.* (Judith HCP)

There was a concern that PWDs’ trust in the eye screening services would be undermined by any changes to screening intervals along with the potential development of DR. In light of previous comments about the embedded nature of the current annual screening programme for PWD, any changes were thought to require careful communication and management.

Additionally, with a potential increase in non-attendance, there were concerns about the length of time a patient would go without being seen in the screening service and the possible impact upon a patient developing DR and the related costs to the NHS.

*What would happen if they DNA if we went onto the two-yearly intervals and they DNAd that two-yearly one? It would be four years then. And that would be more expense wouldn’t it towards the NHS, I think that would cost more because we would have more things going wrong.* (Janine & Hannah HCP)

For PWD and HCP, there were a range of responses to extending screening intervals to 2 years. For some PWD, an extension was welcome as it reflected good diabetes self-care, contrasted with outright rejection for others over concerns about developing eye disease in the extended time period. For HCP, 2-year intervals were acceptable in the context of many patients having minimal or no disease. However, there was some apprehension about the perceptual impact upon patients of changing screening intervals, with PWD feeling that screening was not as important if changed to a 2-year interval.

### Safety of the risk calculation engine (RCE)

Many of our participants (both PWD and HCP) indicated that they would be supportive of the introduction of risk-based allocation to variable screening intervals, on the condition or expectation of particular safeguards or service enhancements being introduced. For HCP, their concerns were focused on the safety of the RCE, specifically, around the quality and availability of data from different areas of health care services (primary and secondary care in the UK) into the RCE and any subsequent allocations into a screening interval being made with incomplete or missing patient data:

*One of my concerns would be getting the right information about the patient from the GPs, from hospitals.* (Sandy & Liz HCP)

This issue of information quality and access was mentioned only briefly by a small number of PWD participants, as might be anticipated given their limited exposure to NHS information systems.

*My only slight concern would be how up to date would the information in the (risk) engine*^1^*if you like would be, in terms of making that decision. And would it be an annual decision that the software made?* (Kevin PWD, Phase 2)

As illustrated above, understanding when the RCE would calculate a PWD screening interval was seen as important. The ISDR RCE calculates the screening interval every time a PWD attends an eye screening appointment; PWD in low and medium risk groups who do not attend are assigned to 12 months for their next invitation and those in the high risk group to 6 months. Some HCP were concerned that the increased complexity of the RCE and subsequent screening allocation could create increased RISK for patients, implying that the mix of data and systems could result in incorrect calculations of risk and allocation to the wrong screening interval.

*It is a more complex system, more complex recipe so there may be more opportunities for it to go wrong."* (John HCP)

The risk engine uses five unconnected data record systems extracted from primary and secondary care, and the screening programme, all with different administrative teams and access/governance arrangements. Data are screened and cleaned through bespoke processing. Risk is then calculated by a chain Markov model using 6 covariates [15].

PWD had similar questions to HCP around the decision-making involved in allocation to screening intervals. They specifically asked about the process of the RCE and how it is constructed.

*Who decides your risks? That’s what I’d like to know.* (Arthur PWD, Phase 2)

In addition, PWD and HCP wanted to be able to self-refer, or refer patients back into annual screening if their ‘risk-factors’ changed between extended screening intervals, as explained below

*I would feel more confident if there were safeguards where I can say, well the nurse can say, oh this is a bit erratic, we will need recourse to the testing place and see if we can get you a quicker appointment… ... in theory if everything stays the same there is no problem with me having the test every three years… so long as there are contingencies in place. If I was confident about safeguards I would be quite happy.* (Derek PWD, Phase 1)

There were calls from PWD for assurances that the recall system would need to mitigate against any diabetic changes which would warrant an earlier recall to eye screening.

*In theory, I would be all right as long as my reading stayed the same. So I suppose if my readings went high, and my sugar levels went high, I could say to the nurse well ok, I've got to have my eyes done now ... If my sugar levels go up for some reason, and I can’t control them you know, I could have my eyes done*. (Jane PWD, Phase 2)

Similarly, HCP wanted assurances that the risk engine would be robust in identifying and inviting all PWD to be screened. Whilst recognising that the RCE was complex and sophisticated, HCP stressed there had to be obvious checks and balances of the system, rather than relying on computers and software.

*There should be some kind of backup where at least there is somebody in the real world who is actually ensuring that things have not gone really haywire.* (Sami & Suzanne HCP)

A further safety concern on extending screening intervals was the potential of missing STDR and the subsequent risk of patients developing visual impairment, as expressed below by a HCP.

*The danger is the longer you leave a recall of course, the more chance you have of missing that occasional patient, so it is cost isn’t, it versus benefits really. And also once you have missed that patient, then trying to deal with them is more expensive.* (Mark HCP)

These concerns were echoed among some PWD who considered that extending eye screening intervals was considered risky as illustrated below.

*How do I know nothing is going wrong in all that time?* (David PWD, Phase 1)

PWD willingness to accept longer intervals between screening episodes was often linked to their general diabetes care, such as regular monitoring of blood sugar levels, and liaising with primary care HCP and eye screening services. So the greater the perceived risk, the less willing PWD were to support an extended screening interval.

### Macro impact of changing screening intervals

Whilst there were many comments about the safety of the ISDR model and its three screening intervals, there were other more wide-ranging comments about the macro effect of changing screening intervals. For example, there was recognition by HCP that the current eye screening system would not be able to manage demands in light of the ever increasing numbers of PWD and the related future cost of screening

*From a burden of health and competing priorities, the NHS finance, we probably would say, there is a recognition that this [risk-based variable-interval screening] is probably for the increasing diabetic population on an annual screen*. (Alice HCP)

Whilst recognising the impact of increasing rates of diabetes on screening, there were some concerns voiced by HCP on their job security with the introduction of variable-interval screening.

*…I think the primary thing everyone is worried about is their jobs. That is, because again we don’t know how many people are going to go to 24 months and how many people are going to go to 6 months, potentially it could you know, cut a lot of people off our list…we are quite concerned about our jobs. (*Janine and Hannah HCP)

There were suggestions that the complexity of the variable screening may serve to disadvantage particular groups of patients. In particular, those groups who do not engage well with services and as a result are at higher risk of developing STDR.

*It will certainly disadvantage this group that we don’t get. We have got to find some way of getting these young, you know, sort of 20s to 40s probably, and a little bit beyond. Because I think you give them an inch that you don’t need them to come for two years – we will never see them for longer.* (Sandy & Liz HCP)

PWD also voiced similar views that risk-based variable-interval screening should enable better targeting of resources and would benefit patients who, for example are difficult to engage and often do not attend for screening, or who are at higher risk of developing STDR.

In the scenario suggested below, there is a recognition that the NHS has finite resources and as such they need to be allocated in a more effective manner.

*We’ll have less demand on the service, therefore we’ll be able to do a better service for other people who need it. That’s my logic. I think it’s sensible to do ... If the evidence shows you that it’s feasible and worthwhile well it just makes sense to refine what you’re doing in a way which is more productive. It doesn’t jeopardise the patient, and it’s a better use of resources which are limited. Makes sense, ticks the boxes, doesn’t it? ... I’m glad it’s happening as a process; it needs to be done.* (Sid PWD, Phase 1)

Other PWD voiced a suspicion that extending intervals between screening episodes for the majority of people with diabetes was financially driven. But instead of being redistributed to be more productive, the cost-savings were aimed at restricting patients’ access to health services.

*I don’t know, if it’s like cost-effective you know, they’re saving money. You feel like they’re saving money to say we don’t want to see you for two years. In your mind you think it’s about the money, otherwise you’d be screening people every six months anyway.* (Ray PWD, Phase 1)

Of note, was that some PWD participants imagined ways in which they could continue to have their eyes screened on a yearly basis, such as staggering other eye appointments, as expressed below.

*If they said you only need it 12 months that will do because I have a second one in the optician anyway*. (David PWD, Phase 2)

Such comments demonstrate misunderstanding about the rationale for different eye appointments and their purpose. Whilst the ISDR model re-calculates a PWD screening interval at every visit, the gaps in understanding of eye screening appointments are a significant issue in supporting PWD to manage all aspects of their diabetes and related care.

*If I go to the optician and I can stagger those visits so one year it’s the diabetes test and the next year it’s the optician’s test, because the optician does look at the back of your eye, then that’ll be ok for me*. (Becky PWD, Phase 2)

As mentioned elsewhere in this paper, there were many examples of confusion amongst PWDs about diabetes, eye disease and eye screening, and, as illustrated above, conflated health beliefs are unhelpful in managing any changes within the eye screening service.

## Discussion

Our findings show general support by PWD and HCP for the introduction of risk-based variable-interval screening. Key factors for our participants were the increasing prevalence of diabetes and finite resources for healthcare. Support was more clearly expressed by HCP, likely related to a better understanding of the aims and current pressures in screening. Our findings are reassuring for policy makers and service providers who are considering introducing variable interval screening, either stratified based on retinal grading or variable intervals based on risk estimation. Our findings also have relevance to other screening programmes with fixed intervals and help to mitigate in part the concerns raised recently in cancer screening [34]. Against these generally supportive findings are a number of important concerns attached to the processes around risk-based allocation and to the screening intervals themselves, clearly expressed by both user groups. These need to be comprehensively addressed to ensure successful implementation, where success can be considered to be early detection of DR, attendance at eye screening and increased understanding of the relationships between diabetes, self-care, and eye screening.

Any changes in health services provision can be problematic for individuals and organisations and can be amplified by routinised practice and behaviours, systemic factors, and local discrepancies in service provision and practice [35–37]. Implementing evidence based practice, such as risk-based variable-interval eye screening, is challenging and needs to address societal, political, cultural, individual and organisational barriers. To take into account all of these variables and their interaction requires a sophisticated theoretical model. Implementation science [38] is emerging as a broad theoretical umbrella with a range of frameworks and strategies, including the BCW. As already mentioned, the BCW identifies three sources of behaviour: capability; opportunity; and motivation. It can then map each of these to appropriate intervention factors for each (and any interactions between them), with a final layer of policy categories to firmly support an intervention.

For example, in our study, both groups of participants expressed concerns about the potential safety of the RCE linking to it being a more complex system, and uncertainty around the reliability of the source data. The BCW allows us to put these concerns into the capability area, and to allay anxieties, there can be a mixture of interventions and policy work. An intervention can be in the form of making transparent the failsafe mechanisms for the RCE to HCP, along with guidelines and policy by the screening programme/Public Health England to ensure that data from primary care practices are embedded with their systems. The use of champions, experts, who are respected in their field, to present and discuss the risk engine to other professionals, where regular updates on the stability and accuracy of variable screening can be fed back to clinicians, can also be a powerful intervention. As we have seen from elsewhere, lessons learnt from the lengthening of screen intervals in other screening programmes, such as breast screening in 2018 [34, 39] show that to address these concerns will require careful explanation of the RCE and transparency about the systems involved. There also had to be opportunities for both HCP and PWD to refer or self-refer back into the eye screening programme, if for example for the latter group, there have been changes in the diabetes severity.

The introduction of the shorter interval for those at greatest risk was widely welcomed by PWD and to a greater extent HCP. PWD perceived the interval as reassuring, while recognising that it meant a higher risk of developing sight threatening disease for them personally. For the former group of PWD, they can be allocated to the motivational area of the BCW, where their emotions around reassurance will probably lead to them attending 6 monthly screening. However, for those PWD who saw a 6 month appointment as high risk, then interventions to encourage attendance would include increased training for HCP on how to approach and engage PWD around monitoring and preventive strategies to lessen the chances of developing STDR. Aligned policy approaches would provide increased service provision for these identified PWD.

The 12 month interval was perceived as standard care, but there were misunderstandings about its purpose allied to an important theme of needing to disentangle health and illness beliefs in PWD around diabetes and eyes, and to develop a clearer understanding of the aims of screening. There continues to be conflation and misconstruction of these concepts along with their interconnectedness and purpose, as previously reported [40–47]. The BCW can usefully place such health beliefs (and potential health behaviours), within all three of its components; capability, opportunity and motivational issues. For example, a PWD who has negative emotions about having diabetes (motivational), will need support (interventions) from HCP, family and friends, perhaps in the form of co-produced information, self-help groups and online forums to increase awareness and confidence in their self-care. In addition, policy changes will be needed to support increased service provision for PWD, not just in primary care but also within communities, such as roadshows. Furthermore, changes to the eye screening services could potentially fracture the trust that PWD have in the current system and in HCP, and by default impact upon diabetes self-care. Thus, introduction of any changes requires thought and consideration, further complicated by the annual screening programme having been in place for over a decade. Old habits die hard, implementing large changes to embedded clinical practice, beliefs and understanding comes with significant challenges and any modifications will require effort by the diabetes care team and the screening programme to explain and consolidate pertinent information.

Potentially, the move to the 24 month interval was controversial for PWD with mixed responses from reassurance to rejection. Most HCP were supportive. Both groups expressed apprehension about the potential impact on care of diabetes by PWD. The BCW suggests that behaviours related to opportunity, such as feeling rejection, is outside of an individual’s control. To allay and soothe such high emotions, which could well lead to non-attendance at screening, can be managed by persuasive relationships (interventions) between a HCP and PWD, where clear explanations are provided that are tailored to the PWD. These conversations can also be backed up with good communication and marketing policy by Public Health England.

Our qualitative study ran in parallel with the ISDR RCT which has shown that risk-based variable-interval screening is safe and effective when compared to annual screening (manuscript under review). Cost-effectiveness is greatly improved allowing resources to be reallocated, including for hard to reach and vulnerable groups. 22% of people declined to participate in the RCT study explicitly stating that they wished to remain in annual screening or did not want a change of interval, this is further evidence of a level of resistance to changing intervals. However dropout in the 2265 PWD randomised to variable interval screening was very low.

### Strengths and limitations

Our PWD participants represented a broad range of individuals with diabetes giving a variety of views from their experiences. Our HCP informants were multidisciplinary and with a range of ages and experience. This affords a breadth of experiences and views, enhanced by our purposive sampling approach and co-produced topic guide. The study was informed by our PI group who commented on all aspects of the research process and were able to inform the content of the topic guides based on their own experiences of diabetes and eye screening.

Our participants may have been motivated to take part in interviews as a way of expressing their views on particular topics related to diabetes care, or gaining social contact. PWD participants in Phase 1 were not the same individuals as in Phase 2.

## Conclusions

Extending intervals and introducing a fully personalised approach is gathering momentum in screening for diabetic retinopathy, and in other areas of disease prevention in the UK.

Our qualitative work sheds new light on the issues around implementing risk-based, variable-interval, and stratified screening using the BCW, with PWD and HCP generally supportive. For successful implementation, a range of issues must be addressed: interpretable and clear safeguards for individual PWD are required against increasing non-attendance, loss of diabetes control and system failures; alternative referral pathways are needed for those lost to follow-up or whose risk factors change substantially over longer intervals; and, for risk calculation systems, reliable monitoring and clear communication is necessary. Utilising the frameworks for changing health services provision in the BCW is likely to improve implementation.

## Data Availability

The data generated and analysed during this study is available from the corresponding author on reasonable request.

## Abbreviations

DR: Diabetic retinopathy
HCP: Health care professionals
PI: Public involvement
PWD: Person with diabetes
RCE: Risk calculation engine
STDR: Sight threatening diabetic retinopathy

## References

[1] https://www.diabetes.org.uk/professionals/position-statements-reports/statistics. Accessed 22 Aug 2019.

[2] https://www.diabetes.org.uk/about_us/news/new-stats-people-living-with-diabetes. Accessed 22 Aug 2019.

[3] https://www.who.int/news-room/fact-sheets/detail/diabetes. Accessed 11 Mar 2019.

[4] Liew G, Michaelides M, Bunce C (2014). A comparison of the causes of blindness certifications in England and Wales in working age adults (16-64 years), 1999-2000 with 2009-2010. BMJ Open.

[5] Agardh E, Tabayat-Khani P (2011). Adopting 3-year screening intervals for sight-threatening retinal vascular lesions in type 2 diabetic subjects without retinopathy. Diabetes Care.

[6] Echouffo-Tcheugui JB, Ali MK, Roglic G, Hayward RA, Narayan KM (2013). Screening intervals for diabetic retinopathy and incidence of visual loss: a systematic review. Diabet Med.

[7] Grauslund J, Andersen N, Andresen J (2018). Evidence-based Danish guidelines for screening of diabetic retinopathy. Acta Ophthalmol.

[8] Looker HC, Nyangoma SO, Cromie DT (2013). Predicted impact of extending the screening interval for diabetic retinopathy: the Scottish diabetic retinopathy screening programme. Diabetologia.

[9] Taylor-Phillips S, Mistry H, Leslie R, Todkill D, Tsertsvadze A, Connock M, Clarke A (2016). Extending the diabetic retinopathy screening interval beyond 1 year: systematic review. Br J Ophthalmol.

[10] Younis N, Broadbent DM, Vora JP, Harding SP (2003). Incidence of sight threatening retinopathy in type 2 diabetes in a systematic screening programme. Lancet.

[11] Basu S, Sussman JB, Berkowitz SA (2018). Validation of risk equations for complications of type 2 diabetes (RECODe) using individual participant data from diverse longitudinal cohorts in the US. Diabetes Care.

[12] Leese GP, Stratton IM, Land M, Bachmann MO, Jones C, Scanlon P (2015). Progression of diabetes retinal status within community screening programs and potential implications for screening intervals. Diabetes Care.

[13] Olafsdottir E, Stefansson E (2007). Biennial eye screening in patients with diabetes without retinopathy: 10-year experience. Br J Ophthalmol.

[14] Pashayan N, Morris S, Gilbert FJ, Pharoah PDP (2018). Cost-effectiveness and benefit-to-harm ratio of risk-stratified screening for breast cancer a life-table model. JAMA Oncol.

[15] Eleuteri A, Fisher AC, Broadbent DM, Garcia-Finana M, Cheyne CP, Wang A, Stratton IM, Gabbay M, Seddon D, Harding SP (2017). Individualised variable-interval risk-based screening for sight-threatening diabetic retinopathy: the Liverpool risk calculation engine. Diabetalogica.

[16] Aspelund T, Thornórisdóttir O, Olafsdottir E (2011). Individual risk assessment and information technology to optimise screening frequency for diabetic retinopathy. Diabetologia.

[17] van der Heijden AA, Walraven I (2014). Validation of a model to estimate personalised screening frequency to monitor diabetic retinopathy. Diabetologia.

[18] van der Heyden AAWA, Ortegon MM, Niessen LW, Nijpels G, Dekker JM (2009). Prediction of coronary heart disease risk in a general, pre-diabetic and diabetic population during 10 years of follow-up: accuracy of the Framingham SCORE and UKDPS risk functions, the Hoorn study. Diabetes Care.

[19] Becker M (1974). The health belief model and personal health behavior. Health Educ Monogr.

[20] Prochaska JO, DiCelemente CC (1983). Stages and processes of self-change of smoking: toward an integrativemodel of change. J Consult Clin Psychol.

[21] Marlatt G, Gordon J (1984). Relapse prevention: a self-control strategy for the maintenance of behavior change.

[22] Michie S, van Stralen MM, West R (2011). The behaviour change wheel: a new method for characterising and designing behaviour change interventions. Implement Sci.

[23] Rubin SE, Davis K, McKee MD (2013). New York City physicians’ views of providing long-acting reversible contraception to adolescents. Ann Fam Med.

[24] Bonner C, Jansen J, Newell BR, Irwig L, Glasziou P, Doust J, McCaffery K (2014). I don’t believe it, but I’d better do something about it: patient experiences of online heart age risk calculators. J Med Internet Res.

[25] Broadbent DM, Sampson CJ, Wang A, Howard L, Williams AE, Howlin SU, Appelbe D, Moitt T, Cheyne CP, Rahni MM, Kelly J, Collins J, García-Fiñana M, Stratton IM, James M, Harding SP, ISDR Study Group (2019). Individualised screening for diabetic retinopathy: the ISDR study-rationale, design and methodology for a randomised controlled trial comparing annual and individualised risk-based variable-interval screening. BMJ Open.

[26] Craig P, Dieppe P, Macintyre S, Michie S, Nazareth I, Petticrew M (2008). Developing and evaluating complex interventions: the new Medical Research Council guidance. Br Med J.

[27] Flick U (2018). An introduction to qualitative research.

[28] Galletta A (2013). Mastering the semi-structured interview and beyond.

[29] Coffey A, Atkinson P (2006). Making sense of qualitative data: complementary research strategies.

[30] Silverman D (2011). Interpreting qualitative data.

[31] Mason J (2017). Qualitative researching.

[32] Smith J, Firth J (2011). Qualitative data analysis: application of the framework approach. Nurse Res.

[33] Gale NK, Heath G, Cameron E, Rashid S, Redwood S (2013). Using the framework method for the analysis of qualitative data in multi-disciplinary health research. BMC Med Res Methodol.

[34] National Health Executive. Backlog of 150,000 cervical screenings revealed as all major health screenings failing to hit targets http://www.nationalhealthexecutive.com/News/backlog-of-150000-cervical-screenings-revealed-as-all-major-health-screenings-failing-to-hit-targets-/220549. Accessed 9 May 2019.

[35] Coleman T (2010). Do financial incentives for delivering health promotion counselling work? Analysis of smoking cessation activities stimulated by the quality and outcomes framework. BMC Public Health.

[36] Grimshaw JM, Shirran L, Thomas R, Mowatt G, Fraser C, Bero L (2001). Changing provider behavior: an overview of systematic reviews of interventions. Med Care.

[37] Summerbell C, Waters E, Edmunds L, Kelly S, Brown T, Campbell K. Interventions for preventing obesity in children. Cochrane Database Syst Rev. 2005:3(5):CD001871–3. 10.1002/14651858.CD001871.pub2 16034868

[38] Bauer MS, Damschroder L, Hagedorn H, Smith J, Kilbourne AM (2015). An introduction to implementation science. for the non-specialist. Psychology.

[39] Duffy SW, Tabar L, Olsen AH, Vitak B, Allgood PC, Chen THH (2010). Absolute numbers of lives saved and overdiagnosis in breast cancer screening, from a randomized trial and from the breast screening programme in England. J Med Screen.

[40] Lewis K, Patel D, Yorston D, Charteris D (2007). A qualitative study in the United Kingdom of factors influencing attendance by PWD diabetes at ophthalmic outpatient clinics. Ophthalmic Epidemiol.

[41] Applebee E (2012). The barriers and enablers that affect access to primary and secondary eye care services — Bradford site report.

[42] Zoega GM, Gunnarsdottir T, Bjornsdottir S (2005). Screening compliance and visual outcome in diabetes. Acta Ophthalmol Scand.

[43] Gray RH, Blades C, Jobson T. Screening clinic non-attendance and the risk of sight threatening retinopathy. Euro J Opthalmol. 2019;19(3):510.

[44] Strutton R, Du Chemin A, Stratton IM, Forster AS (2016). System-level and patient-level explanations for non-attendance at diabetic retinopathy screening in Sutton and Merton (London, UK): a qualitative analysis of a service evaluation. BMJ Open.

[45] Hurrell DD, Donohue S (2012). The barriers and enablers that affect access to primary and secondary eye care services - Glasgow site report.

[46] Hipwell AE, Sturt J, Lindenmeyer A, Stratton I, Gadsby R, O’Hare P (2014). Attitudes, access and anguish: a qualitative interview study of staff and patients’ experiences of diabetic retinopathy screening. BMJ Open.

[47] Moss SE, Klein R, Klein BE (1995). Factors associated with having eye examinations in persons with diabetes. Arch Fam Med.

